# MRI characterization of peripheral arterial chronic total occlusions at 7 Tesla with microCT and histologic validation

**DOI:** 10.1186/1532-429X-17-S1-P404

**Published:** 2015-02-03

**Authors:** Trisha Roy, Garry Liu, Xiuling Qi, Andrew Dueck, Graham A Wright

**Affiliations:** 1 Sunnybrook Research Institute, University of Toronto, Toronto, ON, Canada

## Background

Current treatment guidelines recommend surgical bypass for peripheral chronic total occlusions (CTOs)[1]. Percutaneous transluminal angioplasty (PTA), however, offers a less invasive approach with improved perioperative morbidity, shorter length of hospital stay, and lower cost[2]. Not all lesions are amenable to this technique and there is a significant primary failure rate[2]. Predicting lesion crossability is difficult because current imaging techniques offer limited information with which to characterize CTOs. The ability to distinguish between hard and soft biological substances that compose a CTO can aid procedural planning and facilitate intervention. This pilot study demonstrates the ability of high resolution MRI to characterize peripheral CTO components with microCT and histologic validation.

## Methods

MRI was performed on 10 excised human peripheral arterial CTO segments from 3 patients. Each sample was imaged at 7T (Bruker BioSpec preclinical MR imaging system) at high resolution (75x75x75μm voxels) to produce three-dimensional T2- and T2*-weighted images. For T2-weighted imaging, a spin-echo sequence with an echo-train length of 8 and an equivalent TE of 37 ms was used. For T2*-imaging, an ultrashort echo (UTE) sequence was used with a set of echo times: {20µs, 500µs, 1ms}. A difference image was produced by subtracting the complex signal values of the 1ms-image from those of the 20µs-image. An off-resonance map was produced using linear fits of the phase signals of the set of T2* images. The T2-weighted image, T2*-difference-image, and off-resonance map were used together to differentiate between lesion components. Each sample was imaged with microCT at high resolution (5x5x5μm voxels) to identify calcium. Samples were then decalcified during histologic processing. H&E staining was used to identify microlumina, smooth muscle cells, blood, and residual calcium. Movat's pentachrome staining was used to identify elastic lamina, fibrin, collagen, cholesterol clefts, and proteoglycan.

## Results

**Figure 1:**
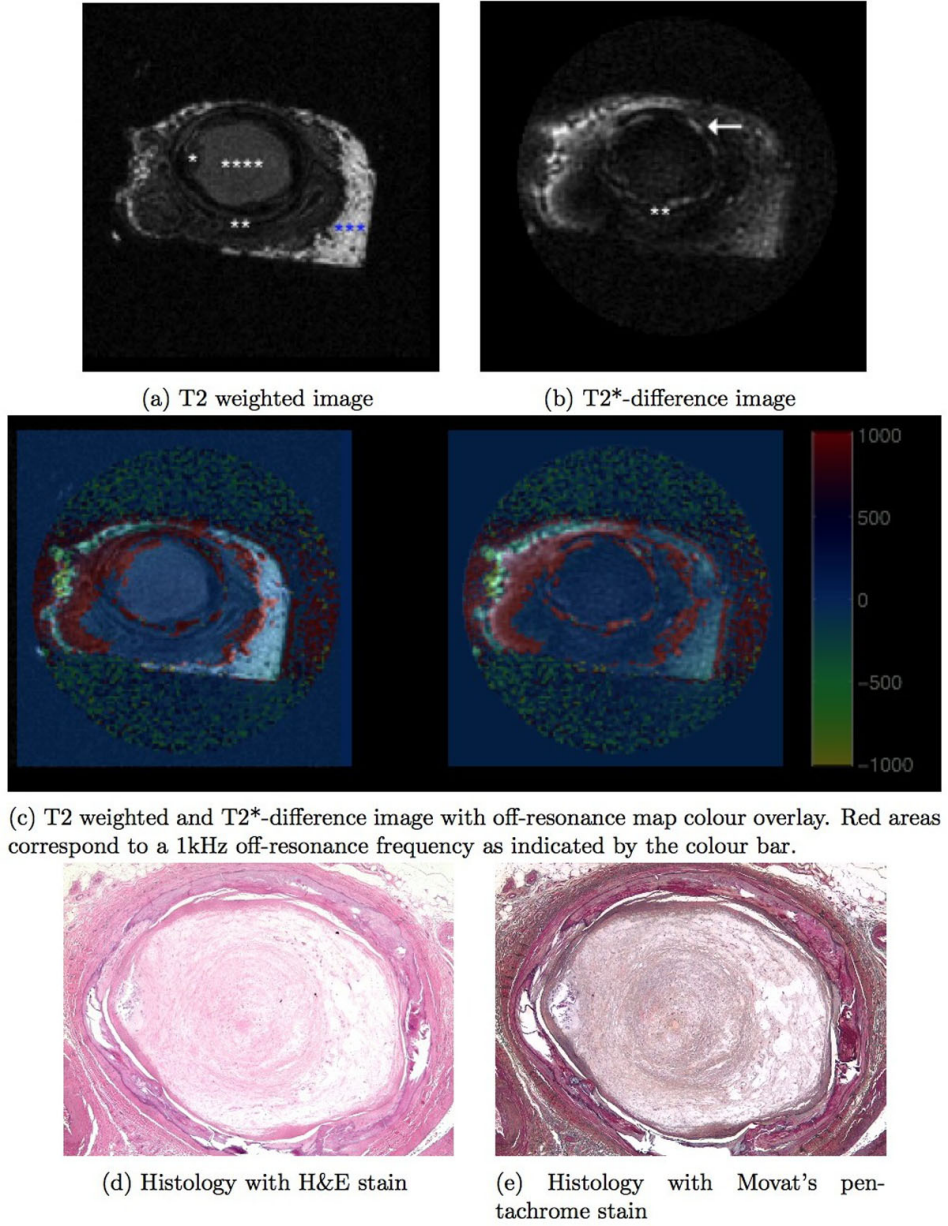
**Human peripheral arterial chronic total occlusion.** The T2 weighted image shows soft lesion components in relative grey scale. Lipid/adipose tissue appears hyperintense (***), loose fibrotic tissue appears grey (****) and cholesterol clefts appear dark grey (*). Hard lesion components like collagen are not seen on T2 weighted images but appears as hyperintense (arrow) on the T2*-difference image. Collagen is also highlighted on the off-resonance map at a 1 kHz off-resonance frequency[4], designated in the colour overlay as red. This collagen imaging signature corresponds to the yellow ring on the Movat's pentachrome stain encircling the CTO core. Calcium is shown as a dark ring (**) on both the T2 weighted and the T2*-difference images.

Hard and soft regions of interest (Table 1) were identified on the T2-weighted, T2*-difference, and off-resonance images with good correlation with microCT and histology (Figure 1).

**Table 1:** CTO components with corresponding imaging signatures

CTO component (in order of descending hardness [3])	T2 weighted	T2*-difference	Resonance shift
Calcium	Black	Black	0-500 Hz

Collagen	Black	Hyperintense	1 kHz

Cholesterol clefts	Dark grey	Dark	0-500 Hz

Loose fibrotic tissue	Light grey	Dark	0-500 Hz

Lipid	Hyperintense	Variable	Variable

Blood/thrombus	Black	Hyperintense	0-500Hz

## Conclusions

These preliminary results demonstrate the potential of high-resolution T2 and T2* imaging using UTE to characterize hard and soft lesion components in human peripheral CTOs. This provides the foundation for further work in determining the lesion crossability and procedural success rates in CTOs.

## Funding

Canadian Institutes of Health Research.
